# Engaging Experts: Science-Policy Interactions and the Introduction of Congestion Charging in Stockholm

**DOI:** 10.1007/s11024-017-9331-3

**Published:** 2017-07-31

**Authors:** Anders Broström, Maureen McKelvey

**Affiliations:** 1 Department of Industrial Economics and Management, KTH Royal Institute of Technology, Stockholm, Sweden; 2 Institute of Innovation and Entrepreneurship, Department of Economy and Society, School of Business, Economics and Law, University of Gothenburg, Gothenburg, Sweden

**Keywords:** Organizational learning, Science-based policy, Evidence-based policy, Interaction, Cognitive distance, Congestion charges

## Abstract

This article analyzes the conditions for mobilizing the science base for development of public policy. It does so by focusing upon the science-policy interface, specifically the processes of direct interaction between scientists and scientifically trained experts, on the one hand, and agents of policymaking organizations, on the other. The article defines two dimensions – cognitive distance and expert autonomy – which are argued to influence knowledge exchange, in such a way as to shape the outcome. A case study on the implementation of congestion charges in Stockholm, Sweden, illustrates how the proposed framework pinpoints three central issues for understanding these processes: (1) Differentiating the roles of, e.g., a science-based consultancy firm and an academic environment in policy formation; (2) Examining the fit between the organizational form of the science-policy interface and the intended goals; and (3) Increasing our understanding of when policymaker agents themselves need to develop scientific competence in order to interact effectively with scientific experts.

## Introduction

An interesting topic of debate in research and public policy concerns the relationships between the domain of science and that of public policy. One foci in this wider debate is the role of scientifically grounded knowledge and the use of scientific results in political processes, such as making public policy decisions. There is a range of different traditions which discuss related topics, but in different ways. Scholarship rooted in political science has engaged with questions such as to what extent policymakers are receptive to scientific knowledge (Landry et al. 2003), and which different functions academic research can play in policy processes (Van de Vall and Bolas 1982; Sunesson et al. 1989). The notion of evidence-based policy has played a central role in such debates, as a contested ideal of transfer of objective evidence from the sphere of science to that of policy (Leicester 1999; Pawson 2002). In contrast, STS scholarship has emphasized the socially constructed nature of the boundary between science and policy. Key contributions to this literature have demonstrated how scientists engage in demarcation activities, which allow the actors to distinguish scientific from non-scientific knowledge (Gieryn 1983). Moreover, this literature has studied how boundaries are renegotiated and reconstructed in the process of exchange between scientifically trained experts and policymakers (Jasanoff 1987).

Similar to scholarship in both strands of literature outlined above, we are interested in the conditions for exchange of knowledge on the boundaries between science and public policy. Our aim is to contribute to this discussion by shifting the focus from the role of scientific knowledge in policy processes to the role of scientific expertise. While codified knowledge in the form of research reviews plays an important role as “evidence-base” for policy in certain fields and circumstances, they are nowhere near to replacing direct interaction between individuals as a medium for science-policy dialogue. The core of this process, we argue, requires the mobilization of scientific competence to inform policymaking processes around a series of specific decisions over time. We hence turn our attention to the question of how scientific experts may contribute to shape policy processes. Specifically, we analyze direct interactions between persons representing scientific knowledge and persons representing policymakers in a policy process which occurs over different phases, and involves different actors.

In this article, we develop a theoretically grounded framework in order to specify the conditions for effective knowledge transfer in the science-policy interface, as well as identify conditions under which problems might arise. Drawing upon organizational learning literature, we propose that the two phases of policy learning and policy implementation place differing requirements on interactions. In particular, we argue that interactions at high or intermediate levels of cognitive distance and with high levels of expert autonomy for policy learning are conducive for policy learning. To effectively support policy implementation, on the other hand, policymaker agents need to find ways to interact with scientific expertise over low or intermediate levels of cognitive distance as well as limited expert autonomy.

The following section reviews existing literature in order to propose a distinction between policy learning and policy implementation as separate modes. It then applies insights from organizational learning and innovation management, in order to identify cognitive distance and expert autonomy as two dimensions which can affect how public policy interacts with science. This literature focuses attention on how an organization can develop strategies to explore and exploit knowledge when making decisions. The section concludes with advancing propositions on how cognitive distance and expert autonomy shapes the conditions for effective policy learning and exchange on policy implementation, respectively. In the subsequent section, this framework is applied to a case study placed in the context of a process to introduce a scheme for congestion charges to reduce traffic in Stockholm, Sweden, in 2006. The case study examines the role of interaction between policymaker agents and scientifically trained experts, specifically scientists working at universities and scientifically-trained consultants to learn about and implement this policy. The final section concludes the article and offers reflections on the organization of the science-policy interface.

## Science and Policy Processes

Public policy is formed through a complex interplay between institutional circumstances, socio-economic changes, stakeholders’ actions and networks (John 1998). Studies on science-policy dynamics have identified the level of political disagreement as a key factor for how scientific expertise and scientific results are used in policy processes. On the one hand, policymakers will engage more intensively with scientific evidence when the issue is contentious, and when it is receiving significant public attention (Lundin and Öberg 2014). On the other hand, political tension also makes policymakers less receptive to adopt their views on the basis of expert advice (Jasanoff 1990; Stevens 2007, 2011). Effective policy learning through the involvement of experts is therefore most likely to happen in policy environments characterized by an intermediate level of political conflict (Weible 2008).

In the present study, we shift the focus away from questions of when and why individuals representing scientific expertise are engaged in policy processes. Our purpose here is to address the far less studied question of how such engagement can be facilitated. Specifically, the present study addresses direct interaction in science-policy interfaces, with a focus on the executive-administrative processes involved with a specific area of public policy. Our analysis thus engages with the conditions for interaction between individuals who in their interaction represent policy and science, respectively. We will refer to the first group as *policymaker agents*, defined as individuals and organizations who are tasked with developing public policy. The latter group is referred to as *scientifically trained experts*. This group is constituted by individuals and organizations with significant science-based knowledge of relevance for the interaction who are *not* employed by organizations responsible for the formulation of policy.

### Learning and Implementation

This article draws upon a distinction between two conceptual modes within a policy process, namely, policy learning and policy implementation. This distinction is inspired by the influential strand of organizational learning literature, which discusses the pursuit of exploration and exploitation in an organization (March 1991). In this literature, we find well-developed conceptual tools for discussing how organizations handle the partly conflicting demands of long-term (exploration) and short-term (exploitation) agendas. Informed by educational psychology and theories about individual learning, organizational learning scholarship constitutes a useful point of reference for our ambition to explore the conditions for direct interaction in science-policy interfaces. In the organizational learning perspective, a central tenant is that exploration and exploitation activities occur iteratively in non-sequential (i.e., non-linear) processes of innovation. This view translates well to how policy-formation processes are described in policy studies (Cerych and Sabatier 1986). In what follows, we develop this parallel between the two literatures further, in order to understand when and where scientific results and scientific competence may be effectively leveraged in policy processes.

In the organizational literature, exploration activities are directed towards developing new types, or a variance of possible activities, and, in other words, are directed to discover what is not yet known. Among such activities are the identification of “problems,” the identification of “solutions,” the matching of solutions to problems (Cohen et al. 1972), and activities of forecasting as well as evaluating the impacts of the adoption of solutions. In the context of policy processes, this article considers that policy learning is equivalent to exploration activities. *Policy learning* refers to a set of activities whereby the general direction of policy is shaped. 1 Furthermore, this concept also includes what Weible (2008) refers to as “political” processes where the direction of policy is formed through the mobilization of stakeholders around a policy decision.

Exploitation activities, in contrast, are designed to apply knowledge and thus the agent implements activities to achieve a well-defined purpose. They also often encompass more incremental changes, e.g., by refining existing activities to enhance characteristics or performance. That is, *policy implementation* refers to activities where the concrete formulation of policy is determined in advance (Sabatier and Mazmanian 1980). For this article, this means activities related to the actual design and implementation of policy, putting overarching policy decisions into practice. This occurs after a purpose has been defined and usually encompasses more incremental changes.

In summary, this article conceptualizes policy learning as exploration activities and policy implementation as exploitation activities. In actual policy processes, these two types of activities generally occur as iterations where policy is formulated, implemented, evaluated, re-formulated, re-implemented and so on (Kingdon and Thurber 1984). For example, in the policy process case study of this article, we identify three phases: a phase leading to the initial idea and identification of the problem and solution (policy learning), a phase of working with the ideas in practice (dominated by policy implementation) and a phase of evaluation featuring elements of both types of activities.

### Cognitive Distance and Expert Autonomy

Another important insight developed within the organizational learning literature is that activities of exploration and exploitation imply different requirements on interaction across organizational boundaries (Colombo et al. 2010). Exploration requires a certain level of knowledge diversity and is often achieved in loosely arranged external partnerships. In contrast, linkages geared towards exploitation are most efficiently set up between partners with significant overlap in terms of knowledge. Moreover, exploitation-oriented interaction activities typically require stricter coordination and hierarchical-type control than exploration-oriented activities do.

In further analogy to the organizational learning literature, we propose that interaction geared towards policy learning and policy implementation, respectively, also require different conditions in terms of knowledge and control opportunities. In particular, we suggest to analyze interaction between policymaker agents and scientifically trained experts in terms of 1) cognitive distance and 2) expert autonomy. More specifically, the article posits that the conditions for policy learning and policy implementation are mediated by cognitive distance and expert autonomy, thereby influencing the outcomes of policy that occur at the science-policy interface. In what follows, we elaborate on how these concepts have been studied in organizational learning literature, and how they translate onto the policy domain.

Cognitive distance, as utilized in the organizational learning literature, is characterized by the degree of separation between two organizations and/or individuals in terms of knowledge bases, values, norms and the heuristics of attribution and decision-making. In general, the cognitive profile of an individual is typically expected to be related to factors such as ethnicity, class, gender etc. (Mohammadi et al. 2017). In the professional context with which we are concerned here, the cognitive distance between two individuals is, however, to a large extent shaped through their work history and educational background (Cummings 2004).

Cognitive distance is relevant, because it has been posited to affect learning. Nooteboom (2000) discusses how the “cognitive distance” between two firms will affect the potential for valuable learning to occur between the firms. At overly large cognitive distances, actors will not be capable of meaningful exchange. However, Nooteboom also argues that too little cognitive distance reduces the value of inter-organizational learning and impulses, in congruence with Bercovitz and Feldman (2011) and March (1991). This literature proposes that there is an inverted U-shaped relationship between cognitive distance and the value of interactions, as an outcome of learning between two organizations. If the organizations are too similar, little learning can occur but if they are too far apart, they will not be able to effectively transfer knowledge either. This argument has been refined and tested for private firms by Nooteboom et al. (2007), who also add the notion that for exploitation activities, cognitive distance creates problems for effective collaboration.

We next consider the role of expert autonomy in exchanges with policymaker agents. Questions about what kinds of control and management arrangements that are best suited for promoting exploration and exploitation, respectively, play an important role in the organizational learning literature. There is significant consensus that there is a fundamental separation between how activities of exploration and of exploitation may be successfully managed. While exploitation activities can be promoted by tightly coupled organizational arrangements, close control will often hamper exploration (Benner and Tushman 2003; Holmqvist 2004). This argument applies to the organization of knowledge creation within an organization, but may be even more accentuated for work across organizational borders. While close managerial surveillance and control over such work may be feasible in settings characterized by explicit knowledge and well-defined objectives (Lam 2000), more loosely coupled arrangements may be necessary for exploratory work.

The second concept that we find useful for studying the conditions for knowledge exchange is therefore *expert autonomy*. This concept is related to the ability of policymaker agents to exercise control over the activities of scientifically trained experts. Expert autonomy can thus range from a low level which can be characterized by close control and surveillance opportunities, to a high level. A high level of expert autonomy would limit the influence of policymaker agents on experts about which questions to ask, the methodology (and in what time horizon) to address these questions, and what conclusions to draw. In an ideal world, a fully autonomous expert such as a university professor without relations to the policymaker agent would have full discretion over all these issues, while an expert with more limited autonomy such as a consultant might base decisions about key aspects of his or her work on openly expressed or indirectly conveyed conceptions about the needs and preferences of policymaker agents.

While the concept of expert autonomy as introduced here has been translated from the organizational learning literature, our suggestion that the degree of autonomy critically shapes how scientifically trained experts contribute to policy processes also resonates well with existing STS scholarship and with some of the literature on co-production of knowledge in particular (cf. Jasanoff 2004). We conceive of knowledge exchange that takes place with anything less than a very high level of expert autonomy as being subject to negotiation between scientific experts and policymaker agents. In other words, the specific knowledge and knowledge artifacts that are being made available for policy processes as a result of direct interaction between scientifically trained experts and policymaker agents is an emergent property of their interaction (Robinson and Tansey 2006). Hence, our characterization of the degree of expert autonomy may be thought of as describing the relative strength of influence of policymaker agents in a process of co-production.

Policy agents’ influence over experts may be exerted in different ways. Many aspects are formal, involving contractual agreements and regulations. Expert autonomy, that is, may be limited as a consequence of direct actions undertaken by the policymaker organization, in order to monitor and ensure control. For example, policymakers may reduce experts’ autonomy by engaging them in an external enquiry – which increases the opportunities for control as compared to interacting with the same expert on a basis of voluntary or advising role. When formulating the conditions of engagement and monitoring an external political enquiry, policymakers will be able to formulate requirements on the scope of the expert’s work and the timing of deliverables.

Moreover, expert autonomy is affected by informal control. Where this is the case, autonomy may be low even in the absence of contractual obligations and active attempts to exert influence. One example is when individual experts and policymaker agents are tied together through an advocacy coalition (Sabatier 1987). Another is when the expert is embedded in an institutional setting that provides incentives to favor interactions with the policy organization. Consultants, for example, who are dependent on future contracts with government agencies may face informal control, as well as researchers at publicly funded research institutes which are expected to demonstrate societal relevance. Dependencies of this kind can lead the experts to internalize policymaker’s interests in their work, even without direct control, and thereby lead to low expert autonomy.

### Inter-Organizational Linkages Between Policymaker Agents and Scientifically Trained Experts

Having established policy learning as exploratory activities and policy implementation as exploitation activities, the previous section on cognitive distance and expert autonomy has focused upon interactions, which are often conceptualized as networks between organizations. Hence, in order to make sense of the concepts of cognitive distance and control at the science-policy interface, the article adopts a network perspective (Borgatti et al. 2009). Specifically, we mean that a focal policy organization is linked to the public science base through a number of one-to-one linkages between the policy organization and external actors such as universities and consultants. Each organization is called a node in network theory. Based on the above analysis, we propose that each linkage between nodes can be characterized in terms of cognitive distance as well as both formal and informal means of control which affect expert autonomy.

Both cognitive distance and expert autonomy are to a large extent determined by organizational level factors, such as the form of interaction (Hunt and Shackley 1999). For example, an independent university researcher may experience a lower degree of cognitive distance when interacting with a specialized government agency than with representatives for central government. As another example, the researcher’s level of autonomy towards the policymaker agent should generally be higher when they are brought in to provide advice on a panel of experts as compared to when they are engaged to undertake specific contract research activities. Furthermore, interaction that takes place though intermediary arrangements such as those referred to by Guston (2001) as boundary organizations may be facilitated so that cognitive distance between scientifically trained experts and policymaker agents is reduced.

Even though the focus of this article is on the organizational level, we do recognize that individual-level idiosyncrasies potentially influence both cognitive factors and control opportunities (Olmos-Peñuela et al. 2015). While the attitudes and cognitive profiles of individuals co-evolve within organizations (Tasselli et al. 2015), individual heterogeneity remains. A policymaker agent and a scientifically trained expert may, for example, have a shared working history, through which they share certain experiences, skills and values. Interaction in any one linkage will be affected by both individual and organizational level characteristics of these organizations and by the characteristics of the involved individuals, as well as the context and organizational form of the interaction.

This article therefore recognizes that our two concepts of cognitive distance and expert autonomy can be constructed as a nexus of the two levels of organizational characteristics and individual-level agency and idiosyncrasy. Similar approaches have been used in analyzing external organizations’ interaction with academic researchers (Broström 2010) as well as in policy studies (Klijn and Koppenjan 2000). In terms of the case study, this implies that we will mainly focus our analysis at the organizational level but will pay particular attention to key individuals linking organizations.

### Propositions Based on Analytical Framework

Based upon the above theoretical reasoning, we develop a set of propositions of how direct science-policy interactions will occur. These propositions are set up as answers to two specific research questions.

#### Under What Conditions Does Interaction Facilitate Policy Learning?

Our proposed answer is that policy learning is facilitated by a linkage which has the characteristic of intermediate cognitive distance. This is based upon the insight that too little cognitive distance will reduce the value of inter-organizational learning and impulses, while at overly large cognitive distances, actors will not be capable of meaningful exchange (as noted for the case of policy-processes by Hunt & Shackley (1999) and Howlett (2009)).

##### **Proposition 1a:**

Policy learning is more effectively facilitated in interactions characterized by an intermediate level of cognitive distance than in interaction characterized by either low or high levels of cognitive distance.

Next, we consider expert autonomy. We first note that there is little need to reduce the autonomy of experts to a low level, since acquiring close control of the activities of scientifically trained experts would not seem to promote learning. Take, for example, the case of interaction in the form of expert hearings. To facilitate policy learning through such activities, policymaker agents must have the means to ensure that selected experts show up at a specific time and place and deliver a statement on a specified topic. There is in general little need to exert strong influence over the experts’ choice of what specific questions to address. In issues of low political tension and genuine uncertainty, such questions are best left to the experts because policymaker agents are not entirely clear about what sort of advice they are in need of. When experts are brought in to give their view on politically contended issues, it can be argued that policy learning – activities through which the general direction of policy is shapes – is only advanced through the expert’s participation when he or she is perceived as acting with a considerable level of autonomy. Only as a representative for “objective” viewpoints can the expert’s participation affect policy formation (Botcheva 2001).

The argument about autonomy as a source of legitimacy would, more generally considered, seem to suggest that policy learning is less likely to take place in interactions where experts are perceived as having a low level of autonomy (Cash et al. 2003). The work of a scientifically trained expert will not provide legitimacy for policy decisions if it is known to have been subject to detailed control. Advice and work from experts known to be in a position of dependency to policymaker agents, or to be part of advocacy coalitions, will be received similarly (Pielke 2007). Hence, we suggest the following proposition:

##### **Proposition 1b:**

Policy learning is more effectively facilitated in interactions characterized by an intermediate or high level of expert autonomy than in interaction characterized by a low level of expert autonomy.

#### Under What Conditions Does Interaction Facilitate Policy Implementation?

Our proposed answer is that a linkage between a policy organization and a scientific partner is most likely to contribute usefully to policy implementation if the linkage is characterized by limited cognitive distance and a limited degree of expert autonomy. A low level of expert autonomy allows policymaker agents to exercise influence over scientifically trained experts, e.g., to focus linkage exchange on a particular problem and to harmonize the timing and content of exchanges with the requirements of the policy implementation process (Stoker and John 2009). A low level of cognitive distance is often necessary to enable communication on the specificities and details involved in policy implementation. Low cognitive distance also implies that policymaker agents and scientifically trained experts have shared understanding of how the specific task at hand in implementation relates to the political situation, to existing regulation, to parallel implementation processes, etc. Thereby, low cognitive distance facilitates relating the subject of exchange to the wider policy context.

##### **Proposition 2a:**

Policy implementation is more effectively facilitated in interaction characterized by a low level of cognitive distance than in interaction characterized by intermediate or high levels of cognitive distance.

##### **Proposition 2b:**

Policy implementation is more effectively facilitated in interaction characterized by a low level of expert autonomy than in interaction characterized by intermediate or high levels of expert autonomy

Table 1 summarizes the propositions as developed above.

**Table 1:** Summary of propositions about what conditions that facilitate policy learning and policy implementation

	Cognitive distance	Expert autonomy
Optimal conditions for policy learning	Intermediate	Intermediate or high
Optimal conditions for policy implementation	Low	Low

## The Case Study: Science-Policy Interactions in the Implementation of Congestion Charges in Stockholm

Our empirical approach was designed to allow for validation of the theoretically grounded framework of how the conditions for policy learning and policy implementation are shaped by cognitive distance and expert autonomy. Furthermore, the case study offered an opportunity to sharpen the constructs and to extend the theoretical framework (Eisenhardt 1989).

Based on our pre-understanding, we identified the implementation of congestion charges as a relevant policy area to study in this context, because we can study interaction between scientifically trained experts and policymaker agents throughout the various modes and phases of a policy process. Furthermore, it offers settings with interaction across both low and high levels of cognitive distance. Scientific research on transportation issues encompasses both highly abstract work on principles as well as application-oriented work seeking to predict changes in travel behavior under alternative implementations of congestion charge schemes. In the latter type of applied work, complex simulation models are developed. Such models would seem to constitute a typical example of an organizational level boundary object; a tangible artifact which provides useful reference points for communication between different groups of actors (Star & Griesemer 1989; Star 2010). In the context of our analytical framework, we may think about the accentuated role of boundary objects such as models and application oriented reports in transportation research as means of reducing the cognitive distance between scientifically trained experts and policymaker agents.

### Context of Congestion Charges in Theory and Practice

Congestion charging as a theoretical phenomenon has received long-standing academic interest. Research on congestion charges can be traced back to the French economist Jules Dupuit, who in his study from 1849 linked the analysis of the social utility of bridge tolls to the reduction of congestion. Later, Pigou (1920) and Knight (1924) incorporated road charging and congestion into social welfare analysis. Notable contributions by Vickrey (1963) and an influential British report (Smeed 1964) spurred further research on congestion charges. This in turn created a strong consensus amongst transport researchers that road charging was necessary to ensure the efficient use of crowded roads, as a complement to the expansion of road networks and public transport externalities, especially during times of peak demand.

In spite of academic support for congestion charging, the actual implementations of charging schemes have been few and far between. In 1975, Singapore became the first city to set up congestion charges (Verhoef 1997), and in 2003, a similar system was introduced in London. At the time of writing, Singapore, London, Milan, Stockholm and Gothenburg are the major cities with general congestion charges as well as a few smaller towns. There is still a strong tradition of analyzing implemented systems, for example, Richards (2006) on the London case.

### Case Study Methodology

In reconstructing the history of the case, we started by utilizing existing written accounts, in particular as offered in Börjesson et al. (2012), Eliasson et al. (2009) and Gullberg & Isaksson (2009). The city of Stockholm also maintains a website about the congestion charges trial (www.stockholmsforsoket.se, in Swedish), from which reports, press material and media coverage of the trial can be reviewed. Although these accounts do provide systematic access to written reports and process events, none of them systematically review our research question, and therefore interviews were necessary. In particular, existing documentation offers limited information which can be used to assess cognitive distance. We therefore interviewed four key persons involved in the process, as identified through the written source material mentioned above. Interviews were held face-to-face and lasted between one and two hours. We also collected secondary data on a wider set of individuals involved in the trial by reconstructing their career histories from CVs and similar material. To ensure reliability, all information from the interviews that we considered using was presented to all interviewees retrospectively. This allowed them to express any objections they might have regarding how we had interpreted their statements, or regarding information we had received from other interviewees.

The analysis of the empirical material was structured around the theoretically derived propositions, as presented above. The coding of the interviews was based on the concepts in Table 1. After having established the structure of the case in terms of the varying intensity of policy learning and policy implementation over time, and identified the most relevant actors, the case analysis focused on assessing cognitive distance and expert autonomy. Data saturation was judged as being achieved when relevant aspects of organizational setups as well as individuals’ employment and education history could be accessed and coherently assessed.

Our assessments of cognitive distance have been primarily based on information relating to the work and education history of individuals. The key assumption that we make is that such experience mold individuals in such a way as to reduce the cognitive distance between individuals with similar professional backgrounds (Hunt & Shackley 1999). For example, a scientifically trained expert who has worked for a transportation agency in the past has developed a certain level of understanding about such work, which lowers the cognitive distance to policymaker agents working in related agencies. A policymaker agent who has been academically trained in transportation analysis has a lower cognitive distance to an active academic in that field than a colleague who has no such training. In interviews, the discussion to capture cognitive distance was structured around questions about the form and purpose of knowledge exchange in various phases, and about the nature of participants’ knowledge, skills and experiences.

Similarly, our assessments of expert autonomy were based on information about organizational affiliation, which was taken from personal career trajectories in general and current employment conditions. A scientifically trained expert who is employed as a consultant in a firm specialized in analysis of transport system is defined as having a limited level of expert autonomy, both in the sense that clients may dictate the conditions for commissioned work and in the sense that the firm is reliant on good relationships with transport authorities for future contracts. A scientifically trained expert employed in academia whose ability to fund his research is not dependent on future commissioned research and who is not connected to policymaker agents through advocacy coalition linkages generally enjoys a high level of autonomy when interacting with policymaker agents. However, we also consider the form of interaction to influence expert autonomy. Policymaker agents could, for example, reduce the autonomy of the above academic to an intermediate level under the arrangement of a commissioned research study or by formally engaging the expert under a temporary contract.

The following sub-sections describe the policy process as occurring in three conceptually separate phases. These phases start sequentially, but become partly overlapping in time.

### Scientific Analysis of Congestion Charging Influences the Policy Debate

The first phase we identified is one involving policy learning as exploration activities. In this phase, which plays out in the period 1990s to early 2000s, scientific analysis influences public debate and an initial pilot trial is started.

Charging drivers for road usage in Stockholm was proposed already in the early 1990s. At this time, the motivation was to obtain financing for additional road projects. In contrast, in the 2006 pilot implementation, the leading political motivation was to reduce traffic by internalizing the costs of congestion externalities. Although the 1990s proposal was never implemented and the idea of road charging was denounced by an influential public investigation due to integrity concerns (SOU 1997), road charging proponents managed to keep the idea alive during subsequent years. Direct interaction between science and public policy was an important factor in this effort, in the sense that the road charging agenda was advanced through a number of investigations into the issue of road charging launched by national and regional public bodies. These governmental bodies usually engaged scientifically trained academics as experts, and they did so by funding research and development activities.

Two notable events involving research activities from the early 2000s can be mentioned. In 2001, the Swedish Environmental Protection Agency (SEPA) commissioned a policy report on road pricing (SEPA 2001). This work came to play a role in generating political attention and in suggesting concrete features for the implementation of road charging (J Dickinson, personal communication, October 17th 2014). Another example is a research project funded by the Swedish Road Administration. In this project, scientifically trained consultants at Transek investigated the effects of different road pricing arrangements on traffic in Stockholm. The simulation models and results from that project made it possible for the firm’s team to respond quickly and with precision when, only months later, the firm was asked to help implement such a system in Stockholm (M Jenstav, personal communication, November 25th 2014).

Both events mentioned above are interesting not only because they demonstrate how scientific competence was mobilized for what we call policy learning purposes, but also because they help identify individuals involved in this phase, who also came to play important roles in science-policy interaction during later phases. Joanna Dickinson, who commissioned the first-mentioned report, later moved from the Environmental Protection Agency to Transek and from there to the policy side. Several other Transek consultants also were engaged in subsequent, more concrete work on the design of a scheme for congestion charging in Stockholm. Our analysis is that such patterns of personal mobility over time decreased cognitive distance between policymaking organizations and scientifically trained experts.

After the September 2002 national and regional elections, there was a round of political negotiations to form a national government as well as a municipal government. In these negotiations, congestion charging came to play an important role. At the municipal level, the Social Democratic party had previously promised voters that they would not introduce congestion charges. The national party organization, however, forced the municipal organization to accept the idea, so that the Social Democratic party and the Green party could build a minority coalition to run the national government.^2^ It was hence decided to develop a full-scale trial, running over several years. In addition to reducing congestion, the Stockholm congestion charge was argued to be a way to finance additional infrastructure projects, and to receive (additional) leveraged funding for desirable local infrastructure projects.

### Scientific Competence Mobilized in Implementation of Congestion Charges

The second phase we identified is dominated by activities geared towards policy implementation in the years 2003 to 2005. However, this phase also includes a process of policy learning in connection with the wider group of stakeholders. Thereby, aspects of exploration and exploitation are combined throughout this phase.

From the very outset of the trial, the implementation of congestion charges in Stockholm was supported and guided by scientifically trained experts. In March 2003, the consultancy firm Transek was asked to draft a full-scale congestion charge system for Stockholm. Dr. Jonas Eliasson had graduated from KTH the Royal Institute of Technology three years earlier, and had written a PhD thesis on transport analysis. He describes his feelings when leaving the first meeting with a group of politicians and officials from the City of Stockholm as follows:

On my way home from the meeting I was in quite a daze. I told my colleagues it felt like one of those scenes in a Hollywood movie when US marines are about to launch an attack, and a deep voice off-camera says to them: ‘This is not an exercise. This is what you have been trained for. Make us proud.’ (Eliasson 2009)

Implementation also required a new organizational structure to support collaboration. Therefore, an “Environmental Charge Secretariat” was appointed that spring, organized under the Stockholm City Council and with a staff of seven people. It started work on June 1^st^, the same day on which the Council made the decision to go ahead with planning for congestion charges. None of the staff had research training, but they did have networks and experience from academic environments, especially in this field of expertise. The head of the secretariat was Magnus Carle, who had 13 years of experience as a part-time assistant teacher in transport planning at the universities focused on engineering, namely, Chalmers and KTH (Royal Institute of Technology). Moreover, Johanna Dickinson, who became responsible for design and evaluation, was recruited from Transek.

The Environmental Charge Secretariat commissioned Transek to come up with a suggestion for toll design and to undertake forecasts of toll impacts on travel behavior. Key reasons for conducting these analyses were to provide a basis for decisions about the location of tolls and about the principles of pricing. Independently of this effort, Stockholm Public Transport (the regional provider of public transport services) undertook a forecast exercise of their own. These forecasts turned out to be quite similar, and in line with assessments made during the trial.

Notably, on a number of relevant issues, the researchers produced somewhat counter-intuitive results, which stood in conflict with what many policymakers thought of as “common sense.” Researchers’ models suggested that in order to achieve the desired effect on congestion, tolls should be exacted not only on cars passing into the charging zone, but also for cars leaving the zone during afternoon rush hours. This principle was adopted in the implementation of the charging scheme, with the addition to extract charges for outbound traffic also in the morning. The latter design twist was a political compromise. It was feared that allowing morning traffic to leave the city free of charge would fuel existing criticism that the charges would only by paid by commuters, whereas the mostly well-to-do citizens in the central city would benefit from traffic reduction without bearing any of the costs.

Another issue, where the scientific analysis was in conflict with prevailing ideas among policymakers, concerned whether the city should be split into two different charging zones or not. Political logic suggested the use of two charging zones, to be able to deal with the above mentioned criticism of charges as disproportionately disadvantageous to suburban commuters. The analysts pointed out that all models used for forecasting suggested that inner-city dwellers would on average pay much more than suburban residents. Furthermore, they showed that a two-zone system, if implemented as suggested by political wisdom, would probably increase rather than reduce congestion. In the planning process that followed, the analysts’ view won out over political logic and was implemented.

Interactions between science and public policy in this phase were primarily mediated through consultants. Although the consultants were scientifically trained, their analysis and forecasts were not produced through novel research. For policy implementation, what was required was satisfactory – not optimal – solutions, and the results needed to be presented in a timely manner (i.e., very hurried, compared to regular scientific activity). Persons involved in these activities suggest that research experience and expertise still proved to be of significant importance. As Eliasson (2009: 215) explains, key members of the consulting staff “had the benefit of being familiar with large numbers of theoretical and model-based studies and investigations,” which enabled them to deliver robust analysis under tight time constraints. Furthermore, the consultancy team was familiar with a number of stylized facts that had been developed through previous research on the public acceptance of congesting charges. In particular, research had demonstrated that charging design and stated objectives must be clearly linked in order for charges to be acceptable to the public.

The start of what we call the policy implementation phase was supported by extensive investigation activity – at one point the secretariat had 20 studies running in parallel (J Dickinson, personal communication, October 17th 2014). In these studies, the scientific expertise that was engaged was almost exclusively that of active consultants. This was a deliberate strategic choice on behalf of the policymakers. Interviews with secretariat staff have confirmed that they had an informal preference for buying in expertise from the open consultancy market rather than directly engaging university faculty. This was the prevailing attitude among the responsible managers, as reported in other research (Gullberg and Isaksson 2009).

Still, a close link with persons with scientific competence from universities also played a role when the actual charging system was designed. A reference group was set up to allow continuous dialogue with key stakeholders (authorities, municipalities, users) on issues such as traffic planning and evaluation design. A number of academic researchers were invited to participate in this group.^3^ Their participation did not leverage specific expertise; the academics were not asked to make further studies and the discussion in the group only rarely touched upon specific results from existing research. However, the participation of academic researchers did considerably facilitate the dialogue between the secretariat and stakeholders (J Dickinson, personal communication, October 17th 2014). They were seen as representing an “objective” perspective on the consequences of congestion charging, and therefore, the organizations involved reported that the presence of academics helped communication amongst all the participants representing different vested interests.

### Scientific Competence Mobilized for Trial Evaluation

The final phase that we identified is characterized by the evaluation of the effects of the congestion charges trial and the subsequent decision to keep or stop the congestion charges. Evaluation activities inherently involve elements of policy learning, but in the case study, the activities involved more development than novel research. A significant share of interaction between policymaker agents and scientifically trained experts during this phase was centered on the execution of evaluation tasks defined by policymakers. As such, the dominant mode of this phase is one of policy implementation, seen as exploitation of known knowledge.

The secretariat initiated the planning for evaluation and assessment of the trial already in November 2003, so parallel with implementation. The secretariat launched a procurement process open only to bidders who could offer scientifically trained competence. Several universities and consultancies submitted tenders. A commission to draw up an assessment strategy for the trial was given to the consultancy Trivector Traffic AB from Lund in southern Sweden. 30 different evaluation projects were executed in 2004–2006, spanning over a wide range of areas. The scope of the projects ranged from studies of travel habits and congestion on roads and in public transport to environmental consequences, effects on retailers and the transportation needs of enterprises, changes in the city environment, attitudes and wider regional economic effects. These were commissioned to several different actors, including university-based researchers as well as consultants.

The ambitious evaluation agenda put a lot of pressure on both the secretariat staff and the experts engaged in carrying out the evaluations. Some of the participating experts and consultants questioned the approach, because there was some overlap between the different evaluations. In retrospect, however, some participants suggest that the evaluations – as well as the respective processes of dialogue which were initiated in connection with them – played important roles in creating acceptance for the trial among the various groups of stakeholders involved (L.S. Rosqvist, personal communication, November 11th 2014). The scientific background of the experts involved in conducting evaluations was from this perspective more important for providing credibility to the process than for guiding concrete decision-making. A reference group was set up in 2004, with representatives from government authorities, municipalities, users and academic researchers.

In parallel, a group of researchers at KTH launched an independent academic study to measure the effects of the trial. This work was funded by national research agencies.^4^ The relationship between the research of the KTH group and the activities commissioned by the secretariat were an issue of some concern for policymakers. There was considerable nervousness on behalf of the secretariat and the regional authority championing the trial about getting criticized by the KTH researchers, of whom a few had a reputation to have a negative position on congestion charging (Gullberg & Isaksson 2009). A few months later, all members of the KTH group were forced to cancel their appointments for the secretariat.

The influence of scientifically trained and academically active individuals on evaluations increased over time during this third phase. At the secretariat, the assessment program was first led by Joanna Dickinsson, who herself was not a trained PhD researcher. Her role was later taken up by Dr. M.B. Hugosson and Lic. Eng.^5^ A. Sjöberg. In January 2005, an advisory group of scientific experts was set up. Their remit was to scrutinize the results of the planning process. Some eight months later, the group became responsible for monthly evaluations. On 1 April 2006, the group – which by now consisted of eight prominent traffic researchers – was given expanded resources and was appointed the task of summarizing all the different evaluations that had been made and also validate their scientific quality.

The trial was launched January 3rd 2006 and terminated July 31st. In the referendum following the trial, Stockholm citizens^6^ voted for the permanent adoption of congestion charges. Several of the researchers who were involved in the evaluation of the trial have over the years published evaluations of the effects of congestion charges, both as research-initiated studies and through commissioned studies (see, e.g., Börjesson et al. 2012; Daunfeldt et al. 2009; Eliasson et al. 2009). The congestion charges are, at the time of writing, still active and the subject of on-going research and debate.

### Analysis of this Case

As described above, scientific research and academic research competence played integral parts in (1) suggesting and motivating congestion charges, (2) in the design of a charging system for Stockholm and (3) in setting up as well as performing multiple evaluations. A key point here is that the nature of interaction between research-trained experts and policymakers differs substantially between the three empirical phases identified above, discussed below in terms of cognitive distance and autonomy.

Table 2 summarizes our analysis of how cognitive distance and expert autonomy in the different phases and sub-phases identified above in the empirical case affected the science-policy interface. Each row represents a (sub-)phase of the process of setting up the congestion charge trial in Stockholm. Columns 2 and 3 contain characterizations of the policy process. Columns 4 and 5 contain assessments of the relationship between scientifically trained experts and policymaker agents.

**Table 2:** Schematic summary of analysis of the congestion charging case in terms of the framework proposed in this article

	Key issue	Dominating mode	Cognitive distance	Expert autonomy
Phase 1	Determining the feasibility of congestion charging	Policy learning	high/intermediate	high
Phase 2: core process	Design of a system for congestion charges in Stockholm	Policy implementation	low	low
Phase 2: stake-holder dialogue	Building acceptance for the implementation of congestion charges among a wider set of stakeholders	Policy learning	intermediate	intermediate
Phase 3: organized evaluation	Designing, performing and summarizing evaluation of outcomes	Policy implementation	low/intermediate	low/intermediate
Phase 3: spontaneous evaluation	Academic research on the effects of the congestion charges trial	Policy learning	intermediate	high

Table 2 illustrates that in phases dominated by learning, interaction between policymaker agents and scientifically trained experts was characterized by high or intermediate levels of expert autonomy and cognitive distance. In phases dominated by implementation, these levels were typically low. This pattern validates the propositions, developed through our analytical framework of how cognitive distance and expert autonomy shape the conditions for policy learning and policy implementation.

In the first phase, the key issue for interaction between policymakers and scientific experts was to determine the political and practical feasibility of congestion charging. In the interactions between them in this phase, both the degree of cognitive distance and of expert autonomy were typically intermediary or large. Academic researchers engaged in abstract modeling of the problem of congestion charges, and hence communication with the policy sphere was primarily indirect. Some indirect interaction was channeled through education, where courses trained future policymakers in models and research results related to congestion charges. Notably, policy-oriented reports also played a facilitating role in keeping the idea of congestion charges on the political agenda because the idea was promoted. This suggests that a reduction of cognitive distance, which was achieved through presenting scientific results in a language and style adapted to the policy context, was instrumental to achieve successful knowledge-transfer.

In the second phase, scientific expertise was engaged in the design of a system for congestion charges in Stockholm. Interactions in this phase took two forms, with interesting differences. The first was in the form of concrete guidance for congestion charges design through directed enquires, on specific issues. Compared to interactions in the first phase, this required lower cognitive distance and a lower degree of expert autonomy. Direct communication between research experts in consultancies and policymakers as well as the ability of the latter to dictate the work of the former was vital to ensure timely and effective interaction of direct relevance to concrete policy decisions (cf. Hunt & Shackley 1999). This phase also, however, saw interaction of a second form. Seeking to build acceptance for the implementation of congestion charges among a wider set of stakeholders, the secretariat also set up a reference group. In this group, scientifically trained experts who were perceived as ‘independent’ were engaged in what seems to have been primarily a facilitating function. The majority of such experts were full-time university professors. In this activity, intermediate to high expert autonomy was required; academic researchers were contributing by representing what was considered independent judgment (cf. Cash et al. 2003). Cognitive distance could in this context be allowed to be relatively high.

In the third phase, scientifically trained experts were engaged by policymakers in the process of designing, performing and summarizing evaluation of the congestion charges trial in Stockholm. In these processes, the participation of researchers with a relatively high degree of expert autonomy (i.e., academically employed researchers) was advantageous. The presence of scientific experts who were being viewed as academic representatives, with some distance between themselves and the political and policy-oriented sphere helped increase the legitimacy of the process for setting up and summarizing the evaluation. Notably, though, there was limited time available for carrying out the individual evaluations, and this created a need to engage experts with limited autonomy (i.e., scientifically trained consultants). Evaluation of congestion charges involves concrete observations and predictions. Scientific abstraction is only involved in outlining cause-and-effect relationships which explain observations. To perform the various evaluations, the expertise required was again found in consultancies with scientifically trained staff, rather than in universities. This ensured that expert autonomy was limited in the sense that a strict and pressed schedule for delivery could be ensured. This arrangement also implied that it was possible to restrict the cognitive distance between experts and stakeholder representatives, whose knowledge about evaluation techniques and the science base on which they draw was mostly very limited.

In order to communicate effectively, research-trained experts and researchers, on the one hand, and policymakers, on the other, were in need of a joint understanding of the situation. The case study has shown that interaction with the policymaker civil servants at the secretariat in this phase required relatively low cognitive distance. As the implementation entered a more evaluation-intensive phase, research-trained staff was recruited to the secretariat, which reduced cognitive distance to the researchers involved in planning and overseeing the evaluation process.

Notably, university-employed academics also engaged in independent research, or what could be called “spontaneous evaluation,” on the effects of the Stockholm congestion charging scheme. Policymakers had no or only indirect influence over the design, methodology or conclusions drawn from such research, which means that the degree of expert autonomy is high. With such research primarily addressing an academic audience, the cognitive distance to the policy sphere is typically higher than in corresponding policy-oriented reports. However, the individuals were then removed from the direct evaluation for policy purposes.

In summary, our analysis of the congestion charges in Stockholm provides additional insights about the relationship between policy learning and expert autonomy in science-policy interaction. We would argue that in the case of scientists communicating research results to policymakers, policy learning can take place in relationships characterized by significant expert autonomy. In other situations, however, scientific competence may need to be mobilized for a specific purpose, e.g., when a policymaking organization identifies a need to learn or to diffuse insights to a wider group of stakeholders with the help of scientifically trained experts. For such purposes, the activities of experts typically need to be adjusted to conditions stated by the policy organization in terms of timing, forms of interaction, etc. to have an impact. The participation of scientifically trained experts in stakeholder dialogue and in policy evaluation may be activities which are most effectively pursued in relationships characterized by an intermediate degree of expert autonomy.

## Conclusions

This article has tackled an intriguing question, namely, under what conditions knowledge exchange between academically trained researchers and civil servants can be expected to impact public policy. This is relevant to many larger debates, such as when and why does effective interaction require that scientists “leave the ivory tower” to engage directly in policy processes? Can we identify circumstances when scientists might waive academic independence in order to bring their expertise to bear on policy-related problems, and yet other sets of problems where it is only by articulating their autonomy that successful knowledge transfer to policy may take place?

More specifically, the article has analyzed the opportunities for knowledge exchange in interactions between scientifically trained experts and policymaker agents. Our analytical framework offers a way to address this issue, by combining two dimensions, drawing from innovation and organizational learning literature. One dimension distinguishes between policy learning (exploration, e.g., activities whereby the general direction of policy is shaped) and policy implementation (exploitation, e.g., activities where the concrete formulation of policy is determined). The second dimension is the degree of cognitive distance combined with expert autonomy. Our propositions are that science-policy interactions at high or intermediate levels of cognitive distance and with high levels of expert autonomy are conducive for policy learning. Policy implementation, on the other hand, requires low cognitive distance as well as limited expert autonomy.

Our analytical framework helps explain why policymakers experience differences in interacting with persons with scientific competencies but working in different types of organizations. By using the concepts of control and expert autonomy, we can help explain science-policy interactions in terms of differences between contract research, independent studies, and temporary forms of interaction such as presenting articles about congestion charging. Hence, the analytical framework can potentially contribute to understanding the role of organizational factors in determining the conditions for science-policy exchange. Over a longer time, diversity of both cognitive distance and expert autonomy is needed, and can be most easily achieved through access to scientifically trained experts with heterogeneous profiles, e.g., to both scientifically trained consultants and university-based researchers with interests and skills in the area.

We used a case study of congestion charges to validate the ideas incorporated in the analytical framework. However, the case study also generated new insights not recognized in our analytical framework. These insights in particular concern how cognitive distance and expert autonomy can be actively managed to facilitate interaction between individuals representing respective organizations of science and policy. One observation is that the establishment of temporary organizations – like the secretariat – may create a network between organizations and thereby facilitate interactions. Previous work on boundary organizations (Guston 2001) has emphasized that the ability to facilitate communication and collaboration between research and policy organizations is contingent on their ability to adapt to changing stakeholder needs (Parker and Crona 2012). Our framework offers more detailed insights into this challenge, in that it highlights the need to carefully manage cognitive distance and expert autonomy. From the perspective of the policymaker organization, the value of the boundary organization as a facilitator of linkages to the public science base will rely on its ability to design various forms of interaction. Such boundary management would consequently imply to manage the differing requirements for policy learning and policy implementation by offering organizational mechanisms that reduces or increases expert autonomy in accordance with current needs, and to mediate contacts to researchers at different levels of cognitive distance from the policymaker organizations.

A second observation from the case study is that individuals’ mobility between organizations may effectively bridge the organizations through their personal networks. More specifically, our interpretation is that contract research and other types of engagements involving temporary job mobility generally allow the two organizations to temporarily move closer together in the short run, and as such, they may have an effect of reducing cognitive distance in the long run as mutual learning takes place. More specifically to the analytical framework, the case study shows how cognitive distance between policymaker agents and scientifically trained experts may be reduced by individual mobility across the (organizational) science-policy divide.

These observations have implications about what may go right, or wrong, for decision-makers in authorities and universities seeking science-policy interfaces. Our view is that the framework facilitates the detection of fallacies in thinking about science-policy interactions. An important one is that if the policymakers expect policy learning to occur through direct interaction with university researchers, this may end in frustration if cognitive distance is too high. An example of when policy learning ambitions may be hamstrung is when policymaker agents are not themselves trained researchers and academics are not sufficiently oriented in the practices of the relevant policy domain. Another example has to do with fundamental epistemological differences, where, say, policymakers seek ‘evidence’ for decision-making while the scientists with whom they interact are not oriented towards producing knowledge recognizable as such. Yet another type of fallacy involves lock-in effects, whereby a certain expert organization that is engaged in interaction with one and the same policymaker organization over time becomes so close to the policy sphere that its ability to provide useful new impulses diminishes. In terms of our framework, we predict that this will happen because repeated interaction reduces cognitive distance and because close ties may leave the expert organization financially dependent on further funding from the policymaker organization. At low levels of both cognitive distance and expert autonomy, the conditions for policy learning deteriorates, which suggests the need for knowledge organizations like universities to maintain autonomy.

This also implies that public policy decision-makers interested in facilitating constructive interaction between science and policy on a specific problem area may need to take action to ensure the availability of scientific expertise, e.g., by monitoring the supply of and, if necessary, supporting PhD education in relevant areas. This follows from the general conclusion that in order to achieve both policy learning and policy implementation objectives, policymakers need to establish both linkages characterized by high or intermediate levels of cognitive distance and expert autonomy, and linkages characterized by low levels in both these dimensions. Public policy may need to work with a diversity of trained scientific experts. We would like to stress that in this case study of congestion charging, public policy has been focused on ensuring the availability of scientific competence. Several persons with PhDs involved in the development of Stockholm congestion were trained in research environments enjoying direct support from national-level authorities with responsibility for transportation policy.

We would like to believe that this article has demonstrated that our understanding of science-policy relationships may be advanced through efforts to promote theoretical generalization at the micro-level of direct interaction between organizations and individuals. STS literature has, on the basis of rich micro-level studies, demonstrated the value of theorizing about macro-level phenomena such as how policymakers and scientists negotiate the science-policy boundary. Literature in the policy studies tradition has provided a rich understanding of under what political conditions scientific advice is likely to impact policy formation. In connecting some of the dots between these literatures, we suggest that organizational perspectives on the opportunities and barriers to effective communication have a valuable role to play.

In terms of future research, we particularly suggest the need to further develop the analytical framework of science-policy interaction through the study of temporary network structures, personal mobility, and of hybrid organizations. Potentially, the two dimensions of cognitive and organizational distance may offer a suitable theoretical basis for the analysis of division of labor within the plethora of organizations from which academically trained experts are engaged in policy processes. Another area for future research is an in-depth investigation of the micro-foundations of cognitive distance and expert autonomy, as well as the further development of techniques to measure these concepts so as to allow across-case comparison.

## Footnotes

1 The term “policy learning” is chosen to connect to the organizational learning literature in an immediate way. Note that while related to the notion of “policy-oriented learning” in the advocacy coalition framework (Sabatier 1987), policy learning as defined here encompasses a wider set of activities and purposes.

2 The decision was taken by the national rather than the local government due to congestion charges being classified as national taxes.

3 Researchers from KTH, LTH, SIKA, GU, the Swedish school of Sport and Health Sciences

4 Vinnova and The Swedish Road Administration.

5 The title Lic. Eng. (Licentiate of Engineering) is in the Swedish university system rewarded for postgraduate research studies in engineering science corresponding to two to three years of full-time studies.

6 Voting was only open for citizens of the municipality of Stockholm. In several other municipalities within the Stockholm County, attitudes towards congestion charges were dominantly negative.
